# Regulation of auxin transcriptional responses

**DOI:** 10.1002/dvdy.139

**Published:** 2019-12-14

**Authors:** Samantha K. Powers, Lucia C. Strader

**Affiliations:** ^1^ Department of Biology Washington University in St. Louis St. Louis Missouri; ^2^ Center for Science and Engineering of Living Systems (CSELS) Washington University in St. Louis St. Louis Missouri; ^3^ Center for Engineering MechanoBiology Washington University in St. Louis St. Louis Missouri

**Keywords:** *Arabidopsis thaliana*, auxin, phytohormone, signal transduction

## Abstract

The plant hormone auxin acts as a signaling molecule to regulate a vast number of developmental responses throughout all stages of plant growth. Tight control and coordination of auxin signaling is required for the generation of specific auxin‐response outputs. The nuclear auxin signaling pathway controls auxin‐responsive gene transcription through the TRANSPORT INHIBITOR RESPONSE1/AUXIN SIGNALING F‐BOX pathway. Recent work has uncovered important details into how regulation of auxin signaling components can generate unique and specific responses to determine auxin outputs. In this review, we discuss what is known about the core auxin signaling components and explore mechanisms important for regulating auxin response specificity.

## Key Findings

- A review of recent updates to our understanding of auxin signaling.

## 1 INTRODUCTION

**Figure 1:**
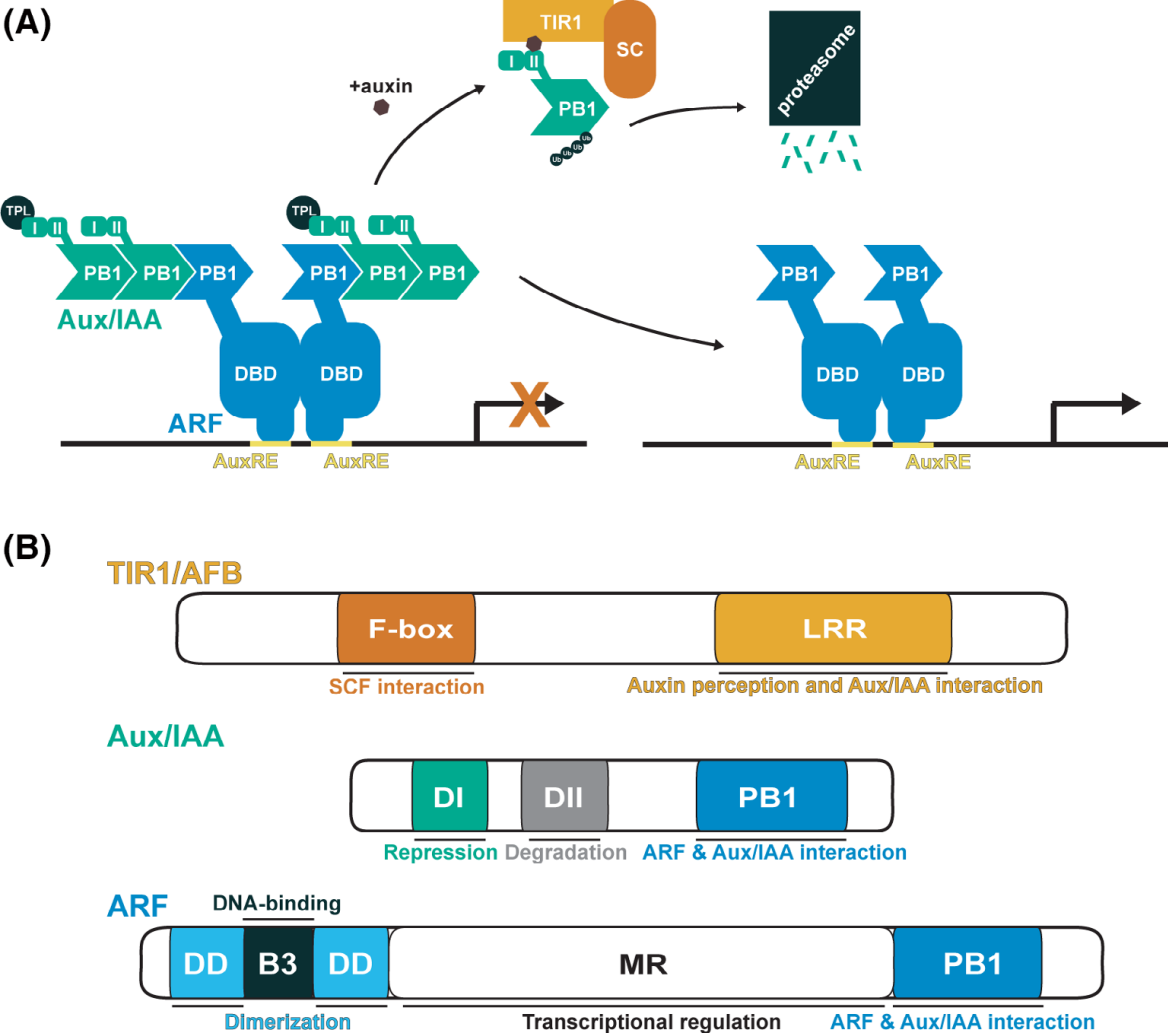
Auxin signaling through the SCF^TIR1/AFB^ pathway. A, In the current auxin signaling model, auxin/indole‐3‐acetic acid (Aux/IAA) repressor proteins interact with and repress auxin response factor (ARF)‐mediated transcription. In the presence of auxin, transport inhibitor response 1 (TIR1) forms a co‐receptor complex with the Aux/IAA and targets it for degradation. Upon degradation of the Aux/IAA, the ARF transcription factor mediates auxin‐responsive transcription. B, Schematic of signaling component domain structures and their role in regulating auxin response

As a principal regulator of growth and development, the phytohormone auxin controls a variety of diverse responses in plants (reviewed in Ref. 1). Nuclear auxin signal perception and consequent alterations in gene expression are carried out by three core auxin signaling components—the TRANSPORT INHIBITOR RESPONSE 1/AUXIN SIGNALING F‐BOX (TIR1/AFB) F‐Box proteins, the AUXIN/INDOLE‐3‐ACETIC ACID (Aux/IAA) repressor proteins, and the AUXIN RESPONSE FACTOR (ARF) transcription factors (reviewed in Refs. 2, 3). In this pathway, the Aux/IAA repressor proteins bind to and inhibit ARF transcription factor activity under low auxin conditions (reviewed in Ref. 4) (Figure 1A). An increase in auxin levels leads to formation of a co‐receptor complex between the Aux/IAA and TIR1/AFB F‐box protein, resulting in ubiquitination and degradation of the Aux/IAA by the 26S proteasome.^4^, ^5^, ^6^ Relief from Aux/IAA repression allows for ARF‐regulated gene transcription (reviewed in Ref. 7).

Despite this seemingly simple signaling pathway, auxin plays a role in regulating a vast array of context‐specific developmental processes throughout the plant. Whereas the large family size of each of these signaling components likely contributes to auxin response specificity, additional factors are involved in generating unique auxin outputs. Here, we review what is known about the core components of the auxin signaling pathway and examine recent advances in our understanding of how these components interact with themselves and additional factors to regulate auxin response.

## 2 AUXIN SIGNAL PERCEPTION BY SCF^TIR1/AFB^

The F‐box protein TIR1 was first identified in a mutant screen for auxin transport inhibitor‐response mutants^8^ and was later shown to function as an auxin receptor.^5^, ^6^ TIR1 belongs to a gene family that contains an additional five AFB proteins.^9^ These six members exist as three pairs of paralogs in the *Arabidopsis* genome: TIR1 and AFB1; AFB2 and AFB3; and AFB4 and AFB5.^10^ Single mutants in *afb1*, *afb2*, and *afb3* display only slight auxin resistance compared to *tir1*, however, higher order mutants result in increased levels of auxin resistance and severe morphological defects.^9^, ^10^ Moreover, AFB1 and AFB2 are unable to rescue the *tir1* mutant, even under expression of the TIR1 promoter.^10^ These results support functional, but unequal roles for these receptors in auxin response and suggest specialized functions for individual family members. Mutants defective in additional components of the SCF^TIR1/AFB^ E3 ubiquitin ligase complex, such as ARABIDOPSIS SKP1 HOMOLOGUE (ASK1), CULLIN 1, and RING‐BOX 1 also display auxin resistance.^11^, ^12^, ^13^, ^14^, ^15^, ^16^ The SCF^TIR/AFB^ E3 ubiquitin ligase is critical for auxin responses.

In addition to the C‐terminal F‐box domain, TIR1/AFB family members contain a N‐terminal leucine‐rich repeat (LRR) domain (Figure 1B). The crystal structure of TIR1 in complex with auxin and an Aux/IAA degron peptide revealed important insights into the mechanism of auxin perception and binding.^17^ The auxin binding pocket lies within the LRR domain, whereas the F‐box domain contacts ASK1.^7^, ^17^, ^18^ With auxin acting as the “molecular glue,” the Aux/IAA peptide interacts with the LRR domain of TIR1 and encloses the hormone‐binding site.^17^

Because the formation of a TIR1‐auxin‐Aux/IAA complex is necessary for auxin binding,^18^ it is possible that different combinations of TIR1/AFB and Aux/IAA proteins may play a role in auxin‐response specificity. Indeed, biochemical studies have revealed binding preferences between these two protein families^10^, ^18^ and recent studies suggest that oligomerization of TIR1 can impact both regulation of Aux/IAA interaction and subsequent degradation rates^19^ and that TIR1 and AFB protein levels are affected by their assembly into a Skp, Cullin, F‐box (SCF) complex.^20^ Further, different co‐receptor TIR1/AFB‐Aux/IAA pairs display unique affinities for auxin^18^ and distinct natural and auxins differentially promote co‐receptor formation^18^, ^21^, ^22^), reflecting differences in the accommodation of the auxin binding pocket in these co‐receptor pairs. Full understanding of biochemical properties driving co‐receptor complex interaction specificity is precluded by a lack of a SCF^TIR1^ structure with a full‐length Aux/IAA. Additional experiments are needed to determine how differences in biochemical properties of TIR1/AFB proteins influence interaction dynamics and auxin response specificity.

## 3 REPRESSION OF AUXIN‐RESPONSIVE GENE EXPRESSION THROUGH AUX/IAA PROTEINS

The *Arabidopsis* genome encodes 29 Aux/IAA repressor proteins that interact with other auxin signaling components through three domains: a degron domain important for interaction with TIR1/AFB proteins (DII), a N‐terminal domain responsible for recruitment of transcriptional co‐repressors (DI), and a C‐terminal type I/II Phox/Bem1 (PB1) domain necessary for interactions with ARF proteins and other Aux/IAA repressors (Figure 1B). Sequence variation within domains among different members of the Aux/IAA family may regulate interaction specificity and therefore auxin output.

### 3.1 Domain II—Degradation

Interaction between DII of the Aux/IAA repressor proteins and TIR1/AFB proteins is required for auxin‐induced degradation of Aux/IAA proteins^18^ and mutations in this domain often result in stabilization of these proteins and decreased auxin responsiveness.^23^, ^24^ Sequence alignments and truncation studies have revealed a 13‐amino acid degron motif within this domain that is necessary and sufficient for auxin‐induced degradation.^14^, ^23^, ^25^, ^26^ Rates of degradation vary among Aux/IAA proteins, with those that have strong matches to the consensus degron motif displaying the most rapid degradation.^27^, ^28^, ^29^ Further, amino acid substitutions within this domain result in altered rates of degradation and aberrant plant phenotypes.^29^ Aux/IAA proteins with a highly diverged degron or completely lacking DII exhibit little to no auxin‐induced degradation,^27^, ^28^ confirming a role for Aux/IAA degradation rates in regulating auxin responses.

Residues outside of DII also contribute to Aux/IAA degradation rates.^27^, ^28^, ^29^, ^30^ A conserved pair of amino acids, a lysine and arginine (KR), is present between domains I and II and play a role in regulating degradation rates; mutations in these residues lead to a significant decrease in auxin‐modulated degradation.^27^, ^29^ Aux/IAA proteins carrying a KQ motif rather than a KR display decreased auxin sensitivity.^27^, ^29^, ^30^ Interestingly, presence of a KR motif does not influence Aux/IAA affinity for TIR1,^29^ suggesting an additional mechanism for increasing efficiency of SCF^TIR1/AFB^ substrate ubiquitination, perhaps by controlling interactions with E2 ubiquitin‐conjugating enzymes.^31^ In addition to the KR motif, a second rate motif was identified immediately C‐terminal to the Aux/IAA degron.^29^ This region is enriched in polar residues and mutation or deletion of this region results in reduced degradation and interaction with TIR1.^29^ In addition to Aux/IAA family members displaying protein‐specific degradation rates, ubiquitination of Aux/IAA proteins can occur both on canonical lysine residues^32^ and noncanonical residues.^33^

The presence of multiple Aux/IAA degradation rate motifs provides deeper insight into mechanisms governing the dynamic nature of this signaling pathway. A recent study using engineered Aux/IAA rate variants demonstrated that lateral root development is strongly dependent on Aux/IAA degradation rate.^34^ Thus, small variations in rate motifs have the potential to generate a tunable system by directing TIR1/AFB‐auxin‐Aux/IAA interactions and Aux/IAA degradation dynamics to trigger specific auxin outputs.

### 3.2 Domain I—Repression

Aux/IAA repression of ARF transcription factor activity is dependent on the recruitment of co‐repressor complexes through interaction with Domain I. DI contains an ethylene response factor‐associated amphiphilic repression (EAR) motif that physically interacts with and recruits Tup1/Groucho/TLE TOPLESS (TPL) and TOPLESS RELATED (TPR) proteins.^35^, ^36^, ^37^, ^38^ TPL/TPR proteins interact with transcriptional regulators from distinct protein families to regulate diverse developmental processes.^35^ Many Aux/IAA proteins interact with TPL/TPR repressors^35^, ^38^ and deletion of DI results in complete loss of repression.^39^ Identical amino acid substitutions in the EAR motif of several Aux/IAA proteins lead to contrasting auxin response phenotypes in plants,^40^ suggesting that sequence‐dictated Aux/IAA‐TPL/TPR interactions may contribute to response specificity.

TPL repression of auxin response genes has been proposed to involve the recruitment of histone deacetylases (HDACs).^37^, ^41^ Histone deacetylation represses transcription by promoting a tight association between histones and DNA, resulting in reduced DNA accessibility.^42^ TPL interacts with HDA19, and both of these proteins are recruited to ARF‐binding sites under low‐auxin conditions.^38^, ^43^ Following auxin‐induced degradation of the Aux/IAA, TPL, and HDAC proteins are removed from these binding sites.^43^ Degradation of the Aux/IAA also unblocks recruitment of SWITCH SUCROSE NONFERMENTING (SWI/SNF) chromatin remodelers^43^ to increase chromatin accessibility at target sites. The existence of multiple mechanisms and levels of repression across Aux/IAA family members may provide an additional source of complexity to the auxin signaling pathway.

Several noncanonical Aux/IAA proteins that lack either the conserved domain II or both domains I and II.^27^ Despite lacking these domains, a recent study uncovered a role for the noncanonical IAA33 in auxin response through interactions with ARF10 and ARF16.^44^ IAA33 negatively regulates auxin response through competition of IAA5 binding to ARF10 and ARF16, thus decreasing repression of these ARF proteins.^44^ Several other noncanonical Aux/IAA proteins, such as IAA20 and IAA30, have been shown to play important roles in plant development.^45^ These findings support a role for noncanonical Aux/IAA proteins in regulating auxin response.

### 3.3 PB1 domain—Oligomerization

Aux/IAA repressors do not have a DNA‐binding domain, but instead repress auxin‐responsive transcription by interacting with the ARF proteins through a C‐terminal PB1 domain present in both protein families (reviewed in Ref. 46). Expression of ARF proteins lacking a PB1 domain leads to constitutive reporter activation in protoplast assays^47^, ^48^ and gain‐of‐function phenotypes in plants^49^ due to loss of Aux/IAA repression. Distinct ARF and Aux/IAA interaction specificities could allow for different auxin outputs and several studies have demonstrated unique binding preferences for varying ARF‐Aux/IAA pairs^46^, ^50^, ^51^; however, the molecular mechanisms underlying interaction specificities have yet to be elucidated.

Some clues into ARF‐Aux/IAA interaction specificity may come from recent structural insight into PB1 domain interaction interfaces. Structural studies have revealed type I/II PB1 domains in ARF^52^, ^53^ and Aux/IAA^54^, ^55^ proteins. Type I/II PB1 domain interactions are driven by opposing electrostatic faces coordinated around two conserved sequence motifs: a positively‐charged invariant lysine and a group of negatively‐charged residues called the OPCA (OPR‐PC‐AID) motif.^54^ The orientation of these distinct faces allows for front‐to‐back interactions between PB1 domains via electrostatic interactions. Mutation of either the conserved lysine or OPCA residues is sufficient to disrupt PB1 domain interactions,^52^, ^53^, ^54^, ^55^, ^56^ however additional, less conserved residues within each of the domain interaction faces also contribute to PB1 domain binding affinity.^3^ Further, recapitulation of the auxin signaling pathway in a synthetic yeast system revealed potential face preference in PB1 domain interactions between ARF and Aux/IAA pairs.^57^ Additional work is necessary to determine if sequence variation in individual PB1 domain faces regulates ARF‐Aux/IAA interaction specificity and whether these pair preferences play a functional role in auxin signaling.

The two‐sided nature of type I/II PB1 domains may allow for protein oligomerization. ARF^52^, ^53^, ^56^ and Aux/IAA^54^, ^55^ proteins multimerize in vitro. Overexpression of a stabilized IAA16 with mutations disrupting either the basic or acidic face of the PB1 domain results in the loss of repressive activity of IAA16,^52^ suggesting that Aux/IAA multimerization is necessary for biological function. However, expression of nonoligomerizing IAA17 and IAA19 in protoplasts had an intermediate effect on repressive activity of these Aux/IAAs^53^; expression of nonoligomerizing IAA1a in Physcomitrella had an intermediate effect on IAA1a repressive activity.^58^ Further, expression of a stabilized nonoligomerizing IAA14 variant efficiently repressed auxin responses,^57^ consistent with IAA14 oligomerization being unnecessary for ARF repression. In addition, Aux/IAA multimerization may be required for efficient recruitment of TPL, as structural studies have shown that binding affinity of the TPL/TPR co‐repressor increases in the presence of oligomerized EAR‐motif containing repressors.^36^ The combination of these data suggests that either (a) multimerization is not necessary for repressor activity of all Aux/IAA proteins or (b) these interactions are more complex than we currently realize. The capacity of PB1 domain‐containing proteins to multimerize adds another potential layer of complexity to Aux/IAA repression of ARF transcription factors, and the biological significance of Aux/IAA oligomerization will need to be examined in further detail.

## 4 ARF PROTEINS REGULATE AUXIN‐RESPONSIVE TRANSCRIPTION

The ARF family of transcription factors drive auxin‐responsive gene expression. Arabidopsis contains 22 full‐length *ARF* genes and one pseudogene (*ARF23*) that cluster into three clades—A, B, and C.^59^, ^60^, ^61^, ^62^ Perhaps unsurprising due to the large number of family members, groups of ARF proteins display some overlap in expression patterns and functional activities.^63^, ^64^ Despite functional redundancy, ARF proteins control a variety of distinct processes during every stage of plant development (reviewed in Refs. 60, 65). There are few confirmed direct ARF targets (Table 1), however, large‐scale DAP‐seq methods have recently identified genome‐wide binding sites of ARF proteins in Arabidopsis^75^ and maize.^76^ Understanding drivers of ARF DNA‐binding specificity will be critical to elucidating outputs of auxin action.

**Table 1:** Direct targets of ARF transcription factors

Gene targets	ARF protein	Function (references)
ATHB8	ARF5	Vascular tissue formation^66^
TMO5	ARF5	Vascular cell division^67^, ^68^
T5 L1	ARF5	Vascular cell division^68^
TMO7	ARF5	Embryogenesis^67^
NTT/WIP4/WIP5	ARF5	Root Initiation^69^
ARR7/ARR15	ARF5	Cytokinin response^70^
TMO3 (CRF2)	ARF5	Cytokinin response^43^
AHP6	ARF5	Cytokinin response^71^
LFY	ARF5	Flower primordium initiation^72^
ANT	ARF5	Cell division and growth^72^
AIL6/PLT3	ARF5	Cell division and growth^72^
FIL	ARF5	Organ polarity^43^
DRN	ARF5	Cotyledon development^73^
LBD29	ARF7	Lateral root initiation^74^
LBD16	ARF7, 19	Lateral root initiation^74^
ARF19	ARF7	Lateral root initiation^74^

The complexity of diverse auxin signaling responses can, at least in part, be regulated by unique characteristics imparted by three distinct protein domains within ARF family members—the N‐terminal DNA‐binding domain (DBD), the variable middle region (MR) associated with activating or repressing activity, and the C‐terminal PB1 domain homologous to those found in Aux/IAA repressor proteins (Figure 1B). Recent studies dedicated to understanding the modular nature of these domains give hints as to how unique auxin outputs can be generated.

### 4.1 DNA recognition and binding by the DBD

Sequence‐specific recognition and binding of the ARF transcription factors to auxin response genes is carried out by the DBD. Structural studies of the ARF1 and ARF5 DBD have revealed a composition of three distinct substructural components.^77^ The first is a plant‐specific B3 domain required for binding of auxin response elements (*AuxREs*) within auxin response genes.^78^ This B3 domain is embedded within a larger second domain, the dimerization domain (DD). The third domain, a Tudor‐like ancillary domain interacts tightly with the DD,^77^ however, a function for this domain is currently unknown.

Several ARF proteins were initially identified based on their ability to bind the canonical TGTCTC *AuxRE* 79, 80 originally found in soybean.^81^ The structure of ARF1 in complex with this element revealed the structural basis for the specificity of DNA binding and identified residues within ARF1 required for mediating this interaction.^77^ Although there is high conservation of these DNA‐contacting residues throughout ARF family members, and multiple ARF proteins bind the TGTCTC motif,^77^, ^80^, ^82^ recent work has identified additional DNA sequences bound by ARF proteins. For example, protein‐binding microarray (PBM) experiments revealed that several ARF DBDs preferentially bind a TGTCTC *AuxRE*.^77^ Further DAP‐seq and computational analysis have revealed other TGTCNN variants that could be involved in auxin response.^75^, ^76^, ^83^, ^84^ Many of these additional *AuxRE* variants must still be validated, however, the presence of multiple target sites with varying ARF binding affinities may begin to help explain differences in ARF activity.

An additional layer of auxin‐responsive gene regulation by ARF transcription factors comes from the ability of these proteins to dimerize through the DD. The DBD of ARF1 and ARF5 form homodimers within the crystal structure with the B3 domains binding to an inverted repeat of the canonical TGTCTC AuxRE element.^77^ Mutation of important dimerization interface residues results in protein unable to replace wild‐type proteins in vivo, confirming the importance of ARF dimerization through the DD in regulating normal biological activity.^77^

Dimerization of ARF proteins allows for multi‐site recognition of auxin responsive motifs within target genes. Mutation of a single *AuxRE* site leads to reduced binding affinity, suggesting that dimerization of ARF proteins leads to cooperative binding at target sites.^77^ As two sites are necessary for high‐efficiency binding, sequence specificity and orientation between binding sites may play a role in determining binding affinity by ARF proteins. Indeed, ARF1 and ARF5 were demonstrated to preferentially bind to *AuxRE*s with different spacing.^77^ These results led to the development of a “molecular calipers” mechanism, in which spacing of auxin responsive motifs, in addition to ARF dimerization, determines transcriptional specificity.^77^ While this model provides an attractive explanation for ARF target specify and response output, further questions remain. For example, are there clearly differentiated binding sites for various ARF proteins in vivo? Do ARF proteins heterodimerize to regulate an even larger number of targets? Answers to these questions will aid in our understanding of how ARF proteins regulate unique auxin response outputs.

### 4.2 Regulation of ARF activity through the middle region

Whereas structural studies of the ARF DBD and PB1 domains have guided our understanding of the function of these domains, much less is known about the properties of the ARF middle region. This region has the most highly diverged amino acid composition and length amongst ARFs and thus it has been difficult to tease apart contributions of the MR in regulating ARF activity. The ARF MR alone is sufficient to confer transcriptional activator or transcriptional repressor activity.^47^, ^85^ These middle regions display amino acid biases, with activation domains enriched in glutamine, serine, and leucine residues, and repression domains enriched in serine, proline, leucine, and glycine residues (reviewed in Ref. 86). This classification of activating or repressing ARF proteins also corresponds to divisions in ARF phylogenetic clades, with all “activator” ARF proteins found in clade A and all “repressor” ARF proteins in clades B and C.^47^, ^59^, ^62^

Although the precise mechanisms that regulate ARF activator and repressor activities are unclear, the MR of ARF proteins likely contain an intrinsically disordered region (IDR) that confers these activities (reviewed in Ref. 65).^87^ Analysis of ARF protein sequences using a disordered prediction algorithm revealed a high degree of disorder in the MR of activator ARF proteins, whereas class B/C ARF proteins do not display strong predicted disorder (reviewed in Ref. 65). Despite lacking folded tertiary structure, IDRs have increasingly been shown to play important roles in protein function by modulating protein interaction and recruitment, affecting DNA binding, or through post‐translational modifications of the region (reviewed in Refs. 88, 89). The ability of IDRs to function in a variety of different contexts could hint at roles for the intrinsically disordered ARF MR in regulating auxin response output; however, further analysis of these regions is needed to establish functions *in planta*.

### 4.3 ARF‐ARF and ARF‐Aux/IAA interactions through PB1 domains

As previously discussed, ARF transcription factor activity can be regulated by interaction with Aux/IAA repressors through PB1 domains. Nearly all Aux/IAA proteins interact with the Class A ARF proteins, however, a limited set of interactions between Aux/IAAs and Class B or C ARFs have been identified.^50^, ^51^ This lack of interaction between Class B and C ARFs and Aux/IAA proteins seems to suggest that the repressor ARF proteins function independently of auxin regulation, and instead compete for DNA binding sites or heterodimerize with other ARF proteins to block transcription.^90^, ^91^ The ability of both activating and repressing ARF proteins to recognize and bind the same *AuxREs* in the promoters of auxin response genes^77^ supports this model; however, repression conferred by the ARF proteins is weaker than repression by Aux/IAA repressor proteins.^90^ Given that auxin responses in the plant must be dynamic and closely regulated, it is possible that multiple modes of repression aid in fine‐tuning auxin responsive gene expression. Clearly additional work is needed to further clarify contributions of repressive ARF activity in regulating gene expression and to determine what part ARF PB1 domains play in these interactions.

Biochemical and structural studies have revealed that ARF PB1 domains multimerize in vitro, in solution, and in the crystal, and that mutations in conserved residues within either the positive or negative face of the PB1 domain are sufficient to disrupt multimerization.^52^, ^53^, ^54^, ^55^, ^56^ Although a biological role for ARF multimerization is lacking, recent mathematical analysis suggests that the ability of ARF proteins to form higher order polymers in solution could play an important role in modulating auxin responses.^92^ Further examination of ARF proteins in vivo is necessary to establish the existence and possible role for ARF multimerization in auxin signaling.

In addition to regulating auxin response through interactions with Aux/IAA repressors, ARF PB1 domain interactions enigmatically play a critical role in DNA binding. Deletion of the ARF PB1 domain results in reduced dimerization of ARF protein DBDs and consequent ability to bind DNA, suggesting that while the DBD is sufficient for ARF dimerization in vivo, interactions through the PB1 domain may act to stabilize DBD dimers.^57^, ^77^, ^82^

Biophysical characterization of PB1 domain interactions between ARF and Aux/IAA proteins showed, for the tested interaction pairs, a preference for ARF‐Aux/IAA heterodimers, with approximately 10‐100‐fold reduction in the affinity for ARF‐ARF and Aux/IAA‐Aux/IAA homodimer self‐interactions.^54^, ^55^, ^56^ Mathematical modeling of the TIR1/AFB, auxin, ARF, and Aux/IAA network provided a conceptual basis for auxin regulation and response driven by these interactions: ARF‐Aux/IAA interactions control response amplitude, Aux/IAA‐Aux/IAA interactions tune speed of the response, and ARF‐ARF interactions regulate specificity^92^ (reviewed in Ref. 93). Thus, interactions driven by the PB1 domain likely play a central role in specifying auxin response.

## 5 EVOLUTION OF AUXIN SIGNALING COMPONENTS

Whereas the complexity and specificity of auxin signaling components have been rigorously studied for several decades, it is only recently that we have begun to try to understand the origin and evolutionary history that imparts this diversity to the nuclear auxin response protein families. Availability of the OneKP transcriptome dataset^94^ has allowed for analysis of multiple species from each major branch of the plant lineage, including algae, bryophytes, lycophytes, ferns, and gymnosperms. Phylogenomic analysis of the core auxin signaling components—ARF, Aux/IAA, and TIR1/AFB proteins, has provided insights into the origin and evolution of these components.

Subdomains of each of these multidomain protein families can be found in red algae and chlorophytes, however, multidomain proteins are only present in charophyte and land plant lineages.^62^ No complete TIR1/AFB or Aux/IAA proteins were identified in charophytes, however, limiting a complete nuclear auxin signaling pathway to land plants.^62^ Several charophytes produce endogenous indole‐3‐acetic acid (IAA)^95^ and show a transcriptional response to exogenous auxin treatment despite the lack of clear auxin signaling orthologues.^62^, ^96^ The mechanism and robustness of auxin response in these species is still unclear and may involve a response mechanism independent of the nuclear auxin response pathway.

ARF transcription factors were established in the common ancestor of green algae and land plants and surprisingly display high conservation of residues important for DNA‐binding.^62^, ^97^ The conservation of ARF proteins and *AuxRE* targets suggests a biologically relevant function for these proteins prior to establishment of TIR1/AFB and Aux/IAA proteins, and presumably consequent auxin‐dependence. Importantly, class A/B ARF proteins diverged from class C ARF proteins in charophytes, likely before the development of auxin‐dependence. This could suggest functions for class C ARF proteins outside of auxin response. Indeed class C ARF display limited interactions with Aux/IAA proteins^50^, ^51^ and the single class C ARF in *Marchantia polymorpha* does not act in auxin‐dependent gene regulation.^62^

The presence of all necessary auxin signaling components can be found in the common ancestor of land plants, however, another important question is how this signaling pathway evolved in complexity to enable diverse auxin responses. *M. polymorpha*, one of the earliest‐diverging land plants, contains a single TIR1/AFB ortholog, a single Aux/IAA, and three ARF proteins (one from each of the three classes) that allow for auxin responsiveness.^98^ Comparative transcriptomics of auxin responsive genes in *M. polymorpha* to those of plant lineages with expanded auxin signaling protein families, such as *P. patens* and *C. richardii*, revealed that the number of ARF transcription factors scales with the number of auxin‐regulated genes.^62^ Further, expansion of the Aux/IAA family likely led to more effective repression of gene activity in the absence of auxin and more tightly regulated auxin response machinery.^62^

Not only have auxin signaling components been examined across evolutionary history,^62^ but the natural variation of these signaling components within *Arabidopsis* accessions have revealed interesting insights. These studies have uncovered evolved sequence variations that correspond to altered molecular phenotypes and underscore how small changes can have significant impacts on protein function and consequent auxin response.^99^ Together, these findings begin to reveal a background for understanding the functions of auxin in plants and how these protein families have evolved and diversified to achieve the high level of complexity seen in the signal response pathway.

## 6 POST‐TRANSLATIONAL REGULATION OF THE AUXIN RESPONSE MACHINERY

Post‐translational modifications of each of the core auxin signaling components impart an additional level of regulation to auxin response outputs (Table 2). For example, S‐nitrosylation of TIR1 modulates auxin responses by enhancing TIR1‐Aux/IAA interaction and promoting Aux/IAA degradation.^100^ *Cis‐trans* isomerization of prolines in Aux/IAA proteins is involved in auxin response, perhaps by regulating recognition by the SCF complex.^101^, ^102^ Also, Aux/IAA proteins interact with and undergo phosphorylation by phytochrome A in vitro.^103^ In addition, ubiquitination of auxin signaling components to target them for degradation may not be limited to Aux/IAA family members, but could also extend to ARF proteins.^107^

**Table 2:** Post‐translational modifications of auxin signaling components

Post‐translational modification	Modified protein	Function (references)
S‐nitrosylation	AtTIR1	Enhances TIR1 interaction with Aux/IAA repressors^100^
cis‐trans isomerization	AtIAA7	Regulates recognition by SCF^TIR1^ 101
	OsIAA11	Promotes Aux/IAA degradation^102^
Phosphorylation	AtIAA3, AtIAA17, AtIAA17, AtIAA1, AtIAA9, PsIAA4	Phosphorylation by Phytochrome A integrates auxin and light signaling^103^
	ARF7/ARF19	Phosphorylation by BIN2 relieves Aux/IAA repression^104^
	ARF2	Phosphorylation by BIN2 reduces DNA‐binding and repressor activity^105^
SUMOylation	ARF7	Regulates ARF7 DNA binding activity to control root branching^106^

Although many of these modifications and their roles in regulating auxin outputs need to be confirmed in vivo, ARF7 and ARF19 are phosphorylated by the BRASSINOID‐INSENSITIVE 2 (BIN2) kinase to regulate lateral root organogenesis.^104^

BIN2‐mediated phosphorylation of ARF7 and ARF19 results in relief from Aux/IAA repression and enhances transcriptional activity by these ARF transcription factors.^104^ ARF2 is also phosphorylated by BIN2 in vitro; however, in this case, ARF2 phosphorylation reduces DNA‐binding and repressor activity.^105^ Finally, several ARF proteins are differentially phosphorylated during maize development,^108^, ^109^ further supporting a role for in vivo regulation of ARF phosphorylation.

In addition, SUMOylation of ARF7 plays a role in hydropatterning of lateral roots.^106^ Differences in water potential triggers modification of ARF7 with the small ubiquitin‐like modifier (SUMO) on the air side of roots. Accumulation of SUMOlyated ARF7 on this side of the root recruits the IAA3 repressor protein and blocks‐auxin responsive gene expression of genes involved in lateral root initiation. Non‐SUMOlyated ARF7 on the opposite side of the root is free to induce expression of ARF7 targets.^106^ Thus, multiple modifications of auxin signaling components (Table 2) can modulate auxin responsiveness.

## 7 ARF CONDENSATE FORMATION TO REGULATE AUXIN RESPONSE

Within the past several years, an increasing number of studies have begun to highlight the importance of biomolecular condensates as a means to regulate diverse biological functions within the cell (reviewed in Ref. 110). These membraneless compartments are driven by phase separation of their components, typically important regulatory or signaling proteins, and are defined by two common features—their ability to concentrate molecules and that they are comprised of biological molecules (reviewed in Ref. 110). Interestingly, recent work has found that some activating ARF proteins form biomolecular condensates in a tissue specific manner within the plant.^87^

A common feature of molecules that form biomolecular condensates is the presence of multiple elements that can regulate intra‐ or inter‐molecular interactions to generate the multivalency needed to drive phase separation.^111^, ^112^, ^113^, ^114^ Both modular protein interaction domains and IDRs play roles in proteins that form biomolecular condensates, as these regions allow for protein assembly into large oligomers or polymers and decrease the solubility of molecules to promote phase separation (reviewed in Ref. 110). Indeed, both the PB1 domain and intrinsically‐disordered MR contribute to ARF condensate formation *in planta*.^87^

Biomolecular condensates affect a wide variety of biological functions including increasing the rate of reaction kinetics, regulating the specificity of biochemical reactions, and sequestering molecules to inhibit activity (reviewed in Ref. 110). ARF condensates are found in the cytoplasm of cells with attenuated auxin responsiveness,^87^ thus ARF condensation likely acts to sequester these transcription factors away from the nucleus to prevent activity. In line with this, disruption of ARF condensate formation leads to massive changes in gene transcription and morphological defects consistent with elevated auxin responses.^87^ Thus, ARF nucleo‐cytoplasmic partitioning and ARF condensate formation are another layer to regulate auxin response.

## 8 ARF COFACTORS AND AUXIN RESPONSE

In addition to ARF‐ARF and ARF‐Aux/IAA interactions, ARF proteins interact with additional cofactors that act as transcriptional regulators (Table 3). At this time, only a limited number of ARF cofactors have been identified; however, it is likely that ARF and Aux/IAA‐interacting proteins could act to further control protein function and specificity. For example, The SWI/SNF ATPases BRAHMA (BRM) and SPLAYED (SYD) directly interact with the middle region of ARF5 to increase chromatin accessibility at ARF5 target loci.^43^ Through regulation of chromatin accessibility, these factors recruit additional transcription factors to ARF binding sites.

**Table 3:** ARF cofactors

ARF cofactors	Interacting ARF protein	Function (references)
BRM/SYD	ARF5	Chromatin accessibility modifications^43^
MYB77	ARF7	Lateral root development and abscisic acid signaling^115^, ^116^
PIF4/BZR1	ARF6	Brassinosteroid signaling^117^
RGA	ARF6,7,8	Giberellin signaling^117^
BPEp	ARF8	Regulates petal growth^118^
BRX	ARF5	Regulates root meristem growth^119^
FUL	ARF6, 8, 2	Promotes fruit valve growth^120^
KAN	ARF3 (ETT)	Ovule development^121^
IND	ARF3 (ETT)	Auxin sensing^122^

The transcription factor MYB77 interacts with both activating and repressing ARF proteins through the PB1 domain, and this interaction is necessary for modulation of several auxin‐inducible genes involved in lateral root development.^115^ MYB77 may also connect abscisic acid signaling and auxin response through interactions with ARF7.^116^ The bHLH protein PHYTOCHROME INTERACTING FACTOR4 and the transcription factor BRASSINAZOLE RESISTANT1 interact with ARF6 through the MR to regulate auxin‐responsive gene expression.^117^ Further, the ARF6 middle region interacts with the DELLA protein REPRESSOR OF GA; this interaction prevents ARF6 binding to target DNA. These findings not only implicate the formation of transcription factor complexes in ARF mediation of auxin response, but also provide a connection between auxin, BR, and GA signaling pathways.^117^ Additional ARF cofactors include the bHLH transcription factor (BIGPETALp) (BPEp) that interacts with ARF8 to regulate petal growth^118^ and the transcriptional co‐regulator BREVIS RADIX (BRX) that interacts with ARF5 to control root meristem growth.^119^ Intriguingly, many of the known ARF cofactors converge on Class A ARF members. Moreover, several of these known interactions seemingly function at the interface of multiple signaling pathways, suggesting that additional signaling pathways can modulate auxin response through interactions with ARF transcription factors.

ARF cofactors may also play an important role in regulating auxin response of atypical ARF proteins, such as ARF3 (ETTIN). ARF3 does not contain a PB1 domain (reviewed in Refs. 86, 123) and therefore likely functions as a noncanonical auxin sensor. Despite this, ARF3 has been shown to interact with the INDEHISCENT (IND) transcription factor to regulate auxin sensing,^122^ as well as with KANADI (Kan) transcription factors to play a role in auxin‐dependent regulation of polarity establishment and organogenesis,^121^ suggesting the importance of ARF cofactors in auxin signaling.

## 9 CONCLUSIONS

Taken at face value, the auxin signaling pathway seems a fairly straightforward mechanism for auxin perception and response; however, we are only beginning to understand the multiple layers of regulation necessary for generating distinct and dynamic auxin outputs. Diversity in signaling component family members allows for different combinations of protein interactions. Additionally, post‐translational modifications, establishment of transcriptional regulatory complexes, and interactions with components from other signaling pathways may play a role in auxin response specificity. Transcriptional regulation of auxin response components,^10^, ^64^ auxin biosynthesis and metabolism (reviewed in Refs. 124, 125), directional auxin transport (reviewed in Ref. 126), and feedback regulation (reviewed in Ref. 127) all contribute additional layers of tunability to this system. Furthermore, other proteins such as SKP2A and IBR5 play a role in auxin response outside of the established TIR1/AFB pathway (reviewed in Ref. 128), suggesting additional levels of auxin response. Integration of all of these factors is necessary to uncover the details involved in the auxin signaling network and advancements in genomic, cellular, computational, and structural studies will surely aid in unraveling the complexity of auxin signaling.

## CONFLICT OF INTEREST

The authors declare no potential conflict of interest.
